# Occurrence of autoimmune pancreatitis after chronic immune thrombocytopenia in a Caucasian adolescent

**DOI:** 10.1007/s12328-021-01383-w

**Published:** 2021-03-20

**Authors:** Hubert Kogler, Wolfgang Novak, Andreas Vécsei, Christina Zachbauer, Wolf-Dietrich Huber, Karoly Lakatos, Katharina Woeran, Judith Stift, Kaan Boztug, Leo Kager

**Affiliations:** 1 Department of Pediatrics and Adolescent Medicine, St. Anna Children’s Hospital, Medical University Vienna, Kinderspitalgasse 6, 1090 Vienna, Austria; 2 Department of Pediatric Nephrology and Gastroenterology, Medical University Vienna, Vienna, Austria; 3 Department of Pathology, Medical University Vienna, Vienna, Austria; 4 Children’s Cancer Research Institute, Vienna, Austria; 5 Ludwig Boltzmann Institute for Rare and Undiagnosed Diseases, Vienna, Austria; 6 CeMM Research Center for Molecular Medicine of the Austrian Academy of Sciences, Vienna, Austria

**Keywords:** Autoimmune pancreatitis, Immune thrombocytopenia, Pancreas, Hematology, Immunology

## Abstract

Autoimmune pancreatitis is a rare, distinct and increasingly recognized form of chronic inflammatory pancreatic disease secondary to an underlying autoimmune mechanism. We report on a 14-year-old boy who developed autoimmune pancreatitis, while he was under treatment with eltrombopag for chronic immune thrombocytopenia. Therapy with corticosteroids resulted in complete remission of both. This is the first report on the co-occurrence of autoimmune pancreatitis and chronic immune thrombocytopenia in childhood, and clinicians should be aware of this rare association, because early diagnosis and therapy of autoimmune pancreatitis may prevent severe complications.

## Introduction

Immune thrombocytopenia (ITP) is an acquired bleeding disorder, caused by autoimmune-mediated destruction of platelets and megakaryocytes [1]. In pediatrics, ITP generally has a good prognosis and is spontaneously resolving in 70–80% of patients. However, in some patients, severe bleedings and a chronic course can occur [1]. In addition, ITP can be complicated by the co-occurrence of other autoimmune diseases, such as autoimmune thyroiditis, celiac disease, systemic lupus erythematosus, autoimmune hepatitis, and other autoimmune cytopenias [2–5].

Within the spectrum of autoimmune diseases, autoimmune pancreatitis (AIP) is increasingly recognized as a distinct entity. AIP is characterized by abdominal pain, obstructive jaundice, pancreatic parenchymal changes caused by lymphoplasmacytic infiltration, and a prompt clinical response to steroid therapy [6, 7]. AIP is extremely rare in children, with only about 50 patients described so far [6, 8]. Of note, ~ 27% of these have been reported to additionally suffer from other autoimmune diseases [6, 7]. However, the co-occurrence of ITP and AIP in childhood has not been reported before.

## Case report

ITP first manifested in the Caucasian male patient at the age of 11 years. He was admitted due to signs of bleeding and laboratory tests revealed severe isolated thrombocytopenia (platelet count 3 × 10^9^/L). Pertinent diagnostic data are provided in Table 1. Except for elevated anti-nuclear antibodies (ANA 1:640, normal negative), no abnormalities were found, and a diagnosis of ITP was made. While repetitive treatments with IVIG (0.8 g/kg) and a short course of corticosteroids resulted in transiently increased platelet counts (> 100 × 10^9^/L), he failed, however, in achieving a stable remission. During each relapse, platelet count was < 10 × 10^9^/L with the presence of cutaneous and/or mucous bleedings. 17 months after diagnosis, treatment with eltrombopag was started at a dosage of 50 mg/day. An initial increase of the platelet count to 144 × 10^9^/L within 3 weeks led to subsequent dose reductions to a final dose of 25 mg/every second day, which resulted in stable platelet counts ranging between 77 × 10^9^/L and 128 × 10^9^/L (median 99 × 10^9^/L).

**Table 1:** Diagnostic work-up and results for chronic immune thrombocytopenia (cITP) and autoimmune pancreatitis (AIP)

Diagnostics	Results (normal values)
Chronic immune thrombocytopenia	
Humoral (auto-)immunity	
* Anti-platelet antibodies*	Negative
* Nuclear antibodies*: ANA, DNA, SM, RNP, RO, LA, SCL, JO, HIST, NUC, ACENA	ANA 1:640 (negative), all others negative
* Antiphospholipid antibodies*: CARG, CARA, B2GPG, B2GPA, B2GPM	Negative
* Mannan binding lectin, complement analyses* (C3, C4)	Normal
* Immunoglobulins:* IgG, IgM, IgA, IgG1, IgG2, IgG3, IgG4	Normal
Cellular immunity (flow cytometry)	
* T-cells*: CD3, CD4/3, CD8/3, CD4/8, HLA-D3, CD25/3, Ta/b, Tg/d, 45RA/45R0, 45R0/45RA, 4/45RA, 4/45R0, 8/45RA, 8/45R0, 45RA/62L/8, 3/127, 3/132, 3/154, DNT	T cells and subtypes: normal
* B-cells*: CD19, IgD + /CD27 + , IgD-/CD27 + , CD21	B cells and subtypes: normal
* NK-cells*: CD56/3	NK-cells: normal
* Lymphocyte function testing:* CD3, SEA and TT induced proliferation	Normal
Bone marrow failure or myelodysplasia/leukemia	
Cytogenetics, diepoxybutan testing	46, XY[10], normal
FLOW	No leukemic cells detectable
FISH-MDS (monosomy 7, trisomy 8, monosomy 5, deletion 5q, deletion 7q)	No MDS typical chromosomal anomalies detectable
Bone marrow analysis (Giemsa staining)	Normal cellularity except for increased number of megakaryocytes, compatible with ITP
Infection diseases	
Virus nucleic acid testing via PCR in bone marrow/plasma: CMV, PVB19, AdV A/B/C/D/E/F/G, EBV	Negative
*Helicobacter* *pylori* (PCR stool)	Negative
HIV serology	Negative
Hepatitis serology: anti-HAV IgG, anti-HAV IgM, HBsAg, anti-HBc IgG, anti-HBc IgM	All negative except for anti-HAV IgG 4 IU/l (negative) and anti-HbSAg > 1000 IU/l
Other autoimmune diseases	
Thyroid hormones (TSH, fT4)	Normal
Celiac disease serology	Normal
Autoimmune pancreatitis	
Pancreas function testing: serum or stool analyses	Amylase 395 U/L (28–100 U/L), lipase 1262 U/L (7–39 U/L), HbA1c 5,4% (4–6%), OGTT normal, elastase < 50 µg/g stool
Liver function testing: serum analyses	GGT 773 U/L (< 52 U/L), ALT 453 U/L (0–31 U/L), AST 247 U/L (0–34 U/L), ALP 947 U/L (< 390 U/l), TBIL 3.1 mg/dl (0–1 mg/dl), BC 2,76 mg/dl (0–0.25 mg/dl), TP 58,7 g/L (60–80 g/L), PRALB 19 mg/dl (12–42 mg/dl)
Tumor marker: Serum analyses	CA 19–9 30.1 kU/L (0–27 kU/L), CEA 0.8 µg/L (0–3.8 µg/L), NSE 19.6 µg/L (0–16.3 µg/L)
Immunology testing: immunoglobulins: IgG, IgM, IgA, IgG1, IgG2, IgG3, IgG4	IgG 548 mg/dl (698–1194 mg/dl), all others normal
Autoantibodies: ANA, DNA, NUC, ENA subsets (RO, LA, SCL-70, SM, RNP, Jo-1, centromer B, c-ANCA, p-ANCA, X-ANCA, smooth muscle, mitochondria, parietal cells, LKM, CARG, CARA, B2GPG, B2GPA, B2GPM, AMA-M2, SP-100, GP210, LC1, SLA	ANA 1:160 (negative), all others negative
Imaging: abdominal ultrasonography	Slightly enlarged liver with normal tissue echogenicity, dilatation of the intra- and extra-hepatobiliary ducts, and a hypoechoic and enlarged pancreatic head
Magnetic resonance cholangiopancreatography	Abrupt termination of the dilated common bile and pancreatic ducts caused by a pancreatic ‘head mass’
Endoscopic ultrasound (EUS)-guided core biopsy-histopathology: HE and immunological staining	Marked fibrosis, lymphoplasmacytic infiltration, and destruction of pancreatic ducts without an increased number of IgG4-positive plasma cells

*ACENA* anti-centromere antibodies, *AdV* adenovirus, *ALP* alkaline phosphatase, *ALT* alanin-aminotransferase, *AMA-M2*: anti-mitochondrial antibodies M2, *ANA* anti-nuclear antibodies, *ANCA* anti-neutrophil cytoplasmic antibodies, *anti*-*HAV* anti-hepatitis A virus antibodies, *anti-HBc* anti-hepatitis B core antigen antibodies, *AST* aspartate transaminase, *BC* conjugated bilirubin, *B2GPA* beta-2-glycoprotein-1 IgA antibodies, *B2GPG* beta-2-glycoprotein-1 IgG antibodies, *B2GPM* beta-2-glycoprotein-1 IgM antibodies, *c-ANCA* cytoplasmic ANCA, *CARA*: anti-cardiolipin IgA antibodies, *CARG* anti-cardiolipin IgG antibodies, *CA19-9* cancer antigen 19–9, *CD3* CD3 positive T-lymphocytes, *CD4/3* CD4 positive T-cell subsets, *CD4/8* CD4/CD8 ratio, *CD8/3* CD8 positive T-cell subsets, *CD19* B-lymphocytes, *CD21* CD21 positive B-cell subsets, *CD25/3* activated CD25 positive T-cell subsets, *CD56/3* CD56 positive CD3 negative NK-cell subsets, *CEA* carcinoembryonic antigen, *CMV* cytomegalovirus, *DNT* TCR alpha/beta positive CD4 negative CD8 negative T-lymphocytes, *DNA* native/double-stranded deoxyribonucleic acid antibodies, *EBV* Epstein–Barr virus, *ENA* extractable nuclear antigens, *FISH-MDS* fluorescence in situ hybridization-myelodysplastic syndrome, *FLOW* flow cytometry, *fT4* free thyroxine, *GGT* gamma-glutamyl transpeptidase, *GP210* anti-glycoprotein-210 antibodies, *HbA1c* hemoglobin A1c, *HBsAg* hepatitis B surface antigen, *HE* hematoxylin and eosin, *HLA-D3* activated HLA-D positive T-cell subsets, *HIST* anti-histone antibodies, *HIV* human immunodeficiency virus, *IgD+/CD27+* IgD positive CD27 positive memory B-cell subsets, *IgD-/CD27+* IgD negative CD27 positive memory B-cell subsets, *JO* anti Jo-1 antibodies, *LA* anti-La antibodies, *LC1* anti-liver cytosol antibodies type 1, *LKM* anti–liver-kidney microsomal antibodies, *NUC* anti-nucleosome antibodies, *NSE* neuron specific enolase, *OGTT* oral glucose tolerance test, *p-ANCA* perinuclear ANCA, *PCR* polymerase chain reaction, *PRALB* prealbumin, *PVB19* parvovirus B19, *RNP* anti-nuclear ribonucleoprotein antibodies, *RO* anti-Ro-antibodies, *SCL* anti-Scl-70 antibodies, *SEA* staphylococcus enterotoxin a, *SLA* anti-soluble liver antigen antibodies, *SM* anti-Smith antibodies, *SP-100* anti-sp100 antibodies, *Ta/b* TCR alpha/beta positive T-lymphocytes, *TBIL* total bilirubin, *Tg/d* TCR gamma/delta positive T-lymphocytes, *TP* total protein, *TSH* thyroid stimulating hormone, *TT* tetanus toxoid, *X-ANCA* atypical ANCA, *3/127* IL-7R alpha-chain positive T-lymphocytes, *3/132* common gamma-chain positive T-lymphocytes, *3/154* CD40L positive T-lymphocytes, *4/45RA* CD4 positive naive T-cell subsets, *4/45R0* CD4 positive memory T-cell subsets, *8/45RA* CD8 positive naive T-cell subsets, *8/45R0* CD8 positive memory T-cell subsets, *45RA/45R*0 CD45RA positive naive T-lymphocytes, *45RA/62L/8* CD45RA positive CD62L positive CD8 positive naive T-lymphocytes, *45R0/45RA* CD45R0 positive memory T-lymphocytes

**Fig. 1:**
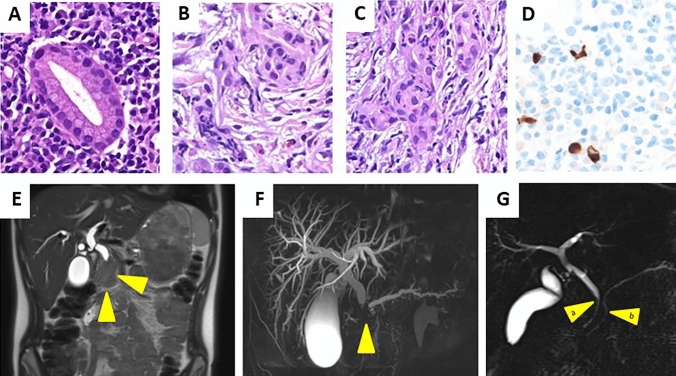
Histopathologic characteristics and imaging findings of autoimmune pancreatitis in a Caucasian adolescent. Depicted are results from pancreas histopathology (**a**–**d**), magnetic resonance imaging (MRI, **e**), and magnetic resonance cholangiopancreatography (MRCP, **f**, **g**). Dense infiltrate of predominantly lymphocytes and plasma cells encases pancreatic ducts (**a**, hematoxylin and eosin (H&E) staining, × 40). Infiltration of the ducts and duct walls by neutrophilic and eosinophilic granulocytes (**b**, **c**, H&E, × 40). Only very occasional in solitary areas IgG4-positive plasma cells (**d**, IgG4 immunostaining, × 40). Frontal T2-weighted abdominal MRI showed enlargement of the pancreatic head (arrow) (**e**). MRCP revealed abrupt termination of the dilated common bile duct (9 mm) and the main pancreatic duct (4.5 mm) caused by a pancreatic head mass (arrow) (**f**). The common bile duct (arrow a) and the main pancreatic duct (arrow b) regained its normal caliber within 2 months of steroid therapy (**g**)

After 25 months on eltrombopag, routine laboratory tests showed elevated transaminases (Table 1). Despite discontinuing eltrombopag treatment, transaminases further increased and he developed icterus and complained of itching and a mild intermittent abdominal pain. Platelet count remained stable after discontinuation of eltrombopag. Further laboratory tests revealed cholestasis and elevated pancreatic enzymes as well as an impaired exocrine pancreas function, whereas endocrine pancreas function remained normal. Pertinent details on further investigations are provided in Table 1. Abdominal ultrasound evaluations revealed dilatation of the intra- and extra-hepatobiliary ducts and a hypoechoic and enlarged pancreatic head. A capsule-like rim surrounding a pancreatic head mass was seen on magnetic resonance imaging (MRI) and magnetic resonance cholangiopancreatography (MRCP) revealed abrupt termination of the dilated common bile and pancreatic ducts caused by the pancreatic head mass (Fig. 1e, f). These findings were suggestive of AIP and endoscopic ultrasound (EUS)-guided core biopsy of the mass with a 22-gauge needle (EZ Shot 3 Plus, Olympus) revealed marked fibrosis, granulocytic infiltration of duct walls, and, in some sections, a dense infiltrate of predominantly lymphocytes and plasma cells encasing pancreatic ducts (Fig. 1a–d), findings characteristic for AIP in childhood [6, 7]. An additional immunohistochemical staining with anti-IgG4 antibody showed scant IgG4-positive plasma cells with one “hot spot” region with 5 IgG4-positive cells/high power field (HPF) and apart from that 0–1 IgG4-positive cells/HPF. Given the adolescent age, the normal serum level of IgG4 (46 mg/dl, reference value 4.9–198.6 mg/dl), and the characteristic histologic findings with granulocyte infiltration of duct walls and only scant IgG4-positive cells, a diagnosis of type 2 AIP was established according to the international consensus diagnostic criteria (ICDC) for autoimmune pancreatitis [9]. After thorough immunological investigations including an NGS-based immunology panel screen to rule out a genetically defined immune defect [10], treatment with oral prednisone (1 mg/kg/day) was initiated and tapered slowly over 4 months. Within 1 month, transaminases, pancreatic enzymes, and cholestasis parameters normalized as well as the follow-up MRCP 2 months after diagnosis. Platelet counts remained stable within the normal range since start of AIP therapy. Regular follow-up investigations are performed (currently every 4 months), and complete remission of AIP and cITP was documented 30 months after discontinuation of steroid therapy.

## Discussion

Herein, we report for the first time the occurrence of AIP after cITP in childhood. Until now, such an association was reported only in 10 Japanese adults (median age of disease onset 68.5 years, range: 61–80 years) [11]. In these Asian adults, AIP and ITP occurred concurrently in two patients; in the other patients, ITP always arose after the onset of AIP [median 4 months (range: 10 days to 4 years)]. AIP was recognized to be IgG4-related in all adults where it was measured. IgG4-related disease is a rare immune-mediated disorder characterized by tissue infiltration by IgG4-positive plasma cells and elevated serum IgG4 [11, 12]. In contrast, our Caucasian patient developed AIP at the age of 14 years and neither had an elevated serum IgG4 level, nor an increased number of IgG4-positive plasma cells in the pancreatic biopsy specimen. This is in line with the previous reports, stating that pediatric AIP (p-AIP) is uncommon as part of IgG4-related disease and that children more commonly follow the disease presentation of type 2 AIP [6–8]. According to the ICDC for autoimmune pancreatitis [9], diagnosis of definitive type 2 AIP in our patient was based on the characteristic histology of idiopathic duct-centric pancreatitis in combination with imaging findings of focal enlargement of the pancreatic head with delayed enhancement and a prompt response to a therapy with corticosteroids. P-AIP, however, is commonly associated with other autoimmune/inflammatory diseases, including Crohn’s disease, ulcerative colitis, glomerulonephritis, and hemolytic anemia; our observation extends the spectrum to cITP [6, 7]. Routine immunological testing (Table 1) including a NGS-based immunology panel screen [10] did not reveal obvious pathologies in our patient’s immune system except for elevated ANAs and a transient mild decrease of IgG. In patients with p-AIP tested for ANAs, up to 40% had detectable antibodies [6–8]. Clearly, further investigations and a thorough follow-up for other potential autoimmune manifestations including systemic lupus erythematodes are required in our patient [13].

CITP was successfully treated with eltrombopag, which is an approved cITP medication [14]. Elevations in liver enzymes or bilirubin are known adverse drug effects (ADRs) of eltrombopag [15]. Thus, monitoring of AST, ALT, and bilirubin is mandatory during therapy. In our patient, the first signs of AIP were elevated transaminases, which further increased after stop of eltrombopag. Therefore, it is unlikely that eltrombopag was the cause of AIP. However, occurrence of pancreatitis has been reported during eltrombopag treatment in at least two adult patients with cITP [16, 17]. The type of pancreatitis was not specified, but careful post-marketing surveillance for the ADR pancreatitis in patients treated with eltrombopag is mandatory.

Complications of p-AIP include failure of exocrine (16% of patients) and endocrine (11% of patients) pancreas function [6, 7]. In our patient, exocrine pancreas insufficiency was fully reversible, and early diagnosis and therapy as reported herein may have contributed to this favorable outcome.

Clinicians should be aware of the co-occurrence of cITP and p-AIP. Abdominal pain and obstructive jaundice are suggestive for p-AIP. Imaging studies and laboratory investigations can help to quickly establish the diagnosis, and therapy with steroids typically induces remission.
